# Elevated amylase in plasma represents an adverse prognostic marker in patients with metastatic pancreatic cancer: A retrospective analysis

**DOI:** 10.1007/s00508-018-1383-3

**Published:** 2018-08-21

**Authors:** Eva Asamer, Joanna Szkandera, Paul Gibiser, Anna Lena Lembeck, Tatjana Stojakovic, Peter Kornprat, Caroline Lackner, Thomas Winder, Konstantin Schlick, Herbert Stöger, Armin Gerger, Martin Pichler, Michael Stotz

**Affiliations:** 1 Division of Clinical Oncology, Department of Medicine, Medical University of Graz, Auenbruggerplatz 15, 8036 Graz, Austria; 2 Clinical Institute of Medical and Chemical Laboratory Diagnostics, Medical University of Graz, Graz, Austria; 3 Division of General Surgery, Department of Surgery, Medical University of Graz, Graz, Austria; 4 Institute of Pathology, Medical University of Graz, Graz, Austria; 5 Department of Oncology, University Hospital Zurich, Zurich, Switzerland; 6 3rd Medical Department with Hematology and Medical Oncology, Hemostaseology, Rheumatology and Infectious Diseases, Paracelsus Medical University, Salzburg, Austria; 7 Research Unit Genetic Epidemiology and Pharmacogenetics, Division of Clinical Oncology, Department of Medicine, Medical University of Graz, Graz, Austria; 8 Department of Experimental Therapeutics, The University of Texas MD Anderson Cancer, Houston, TX USA

**Keywords:** Lipase, CA19‑9, Pancreas, Pancreatitis, Adenocarcinoma

## Abstract

**Background and aim:**

The aim of this study was to investigate the prognostic relevance of plasma amylase and lipase concerning survival of patients suffering from metastatic pancreatic cancer (PC).

**Method:**

This retrospective study included 351 patients with metastatic PC, who were treated in a single academic institution. Cancer-specific survival (CSS) was analyzed using the Kaplan-Meier method. To further evaluate the prognostic significance of lipase and amylase, univariate and multivariate values were calculated using Cox proportional models.

**Results:**

In univariate analysis, an increased amylase level was associated with shorter CSS in PC patients (hazard ratio HR = 1.258; 95% confidence interval CI = 1.011–1.566; *p* = 0.039). In multivariate analysis, including gender, age, CA19-9 and administration of chemotherapy, increased amylase levels prevailed as an independent prognostic factor for CSS (HR = 1.373; 95%CI = 1.004–1.878; *p* = 0.047).

**Conclusions:**

Plasma amylase was found to be an independent prognostic factor in patients with metastatic PC. The results indicate that amylase might represent a novel and useful marker for better patient stratification in PC management.

## Background

Pancreatic cancer (PC) is currently associated with the lowest survival rates among all known malignancies, a fact that has hardly changed over the last decades. With over 53,670 new cases each year and 43,090 PC-related deaths, the demand for a better understanding of this fatal disease becomes all the more evident [1]. During the last decade, many studies have been conducted in order to improve the overall survival rate of PC but the only potential chance for a cure remains a total resection [2]; however, merely 15–20% of patients can be considered for surgical resection and pancreatectomy as surgery can only succeed if the tumor is still in its early stages and has not formed distant metastases [3]. Unfortunately, at the time of diagnosis, the majority of patients present with either locally advanced or metastatic disease [4, 5]. To predict the duration of patients’ clinical outcome, only a small variety of parameters, apart from radiological examinations, are frequently employed. Numerous studies, investigating the tumor marker CA19-9, have shown that there is a correlation between the level of CA19-9 and the survival rate of patients with advanced disease [6, 7]. Other laboratory markers including the systemic inflammatory response have been extensively studied and were proposed as useful in the prognosis of PC patients [8–11].

The question posed in this study was whether the pancreatic enzymes lipase and amylase in plasma can be used as prognostic predicators as well. This is especially significant as determining blood-based marker is a non-invasive and gentle method towards cancer patients. The enzymes addressed in this study are commonly tested for assessment in suspected acute pancreatitis but their significance with respect to cancer patients is hardly considered [12].

Currently, there are no data for PC regarding amylase and lipase as prognostic markers. Therefore, the aim of the present study was to explore whether measured amylase and lipase levels can lead to a statistically significant improvement of the prognosis.

## Material and methods

This retrospective analysis included data from 351 patients with metastatic PC (stage IV), who were treated at the Division of Clinical Oncology, Medical University of Graz, from January 2004 to October 2015. All patients presented with histologically confirmed ductal adenocarcinoma of the pancreas and available clinical records including the plasma levels of amylase and lipase. These pancreatic enzymes were determined routinely in the prediagnostic workflow. Patients with other histological subtypes of PC (e.g., azinar, neuroendocrine or mucinous-cystic neoplasms) were excluded from the study as they have varying clinical outcome [13]. For deceased patients, dates of death were obtained from the central registry of the Austrian Bureau of Statistics, clinical records or telephone calls. Patient records and information were anonymized and de-identified prior to analysis. All clinicopathological data were retrieved from medical records at the Division of Clinical Oncology, as well as from pathology records from the Institute of Pathology at the same institution. Since the TNM classification system for PC changed during the study period, tumor stages were uniformly adjusted according to the 7th edition of this system. Other documented clinicopathological parameters included administration of chemotherapy, gender and age. The levels of pancreatic enzymes were determined in heparinized plasma within 1–3 days prior to the histologically proven diagnosis (by needle biopsy) and performed as a part of routine clinical practice. Lipase and pancreatic amylase were measured enzymatically (Roche Diagnostics, Mannheim, Germany) on a Cobas analyzer from Roche.

Treatment decisions were made according to the current European Society for Medical Oncology (ESMO) guidelines at the time of treatment. The study was approved by the local ethical committee of the Medical University of Graz (26-196 ex 13/14).

Cancer-specific survival (CSS) was defined as the time span from the date of histologically proven diagnosis to cancer-related death (measured in months). The optimal cut-off values for amylase and lipase were determined by applying receiver operating curve (ROC) analysis to differentiate between survival and death (using the MedCalc statistical software version 13.3) as previously described [14]. The association between the pancreatic enzymes and clinicopathological parameters were evaluated through parametric or non-parametric tests (Student’s T‑test, Mann-Whitney U-test and χ^2^-test). Patients’ clinical endpoint was calculated using the Kaplan-Meier method and tested for significance using the log rank test. Univariate and multivariate Cox proportion analysis were performed to determine the influence of different clinicopathological parameters on CSS. Hazard ratios (HR) estimated from the Cox analyses were recorded as relative risks with corresponding 95% confidence intervals (CI). All statistical analyses were performed using the Statistical Package for Social Sciences version 20.0 (SPSS Inc., Chicago, IL, USA). A two-sided *p* < 0.05 was considered statistically significant.

## Results

Within the study cohort, patients had a median age at diagnosis of 66.0 years (interquartile range 57–72 years, minimum 35 years, maximum 85 years) Median levels of CA19-9 were 1191.7 U/ml (interquartile range 177–10,390 U/l; normal range in healthy individuals: 0–37 U/l). Median survival time was 6 months (range, 0–44 months) and 338 (96.3%) of the patients had died by the date of the last follow-up. The mean value determined for amylase was 35 U/l (range 1–561 U/L, normal range in healthy individuals: 15–53 U/l) and 95 U/l for lipase (range 5–1597 U/l; normal range in healthy individuals: 13–60 U/l). An overview of all clinicopathological parameters in this study cohort is shown in Table 1.

**Table 1:** Frequency of clinicopathological parameters in patients with stage IV pancreatic cancer in this cohorts

Parameter	Number (%)
*Gender*	
Female	152 (43.3)
Male	199 (56.7)
*Age (years)*	
<65	147 (41.9)
≥65	204 (58.1)
*Karnofsky index*	
<90	221 (63)
90–100	130 (37)
*Lipase*	
<36 U/l	167 (47.6)
≥36 U/l	184 (52.4)
*Amylase*	
<20 U/l	165 (48.2)
≥20 U/l	177 (51.8)

**Fig. 1:**
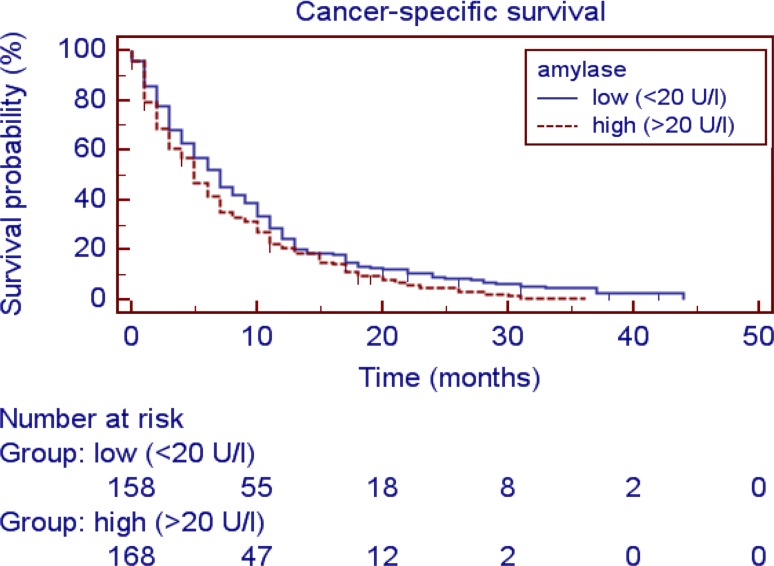
Kaplan Meier curve: Cancer specific survival: elevated Amylase vs. non elevated Amylase

**Fig. 2:**
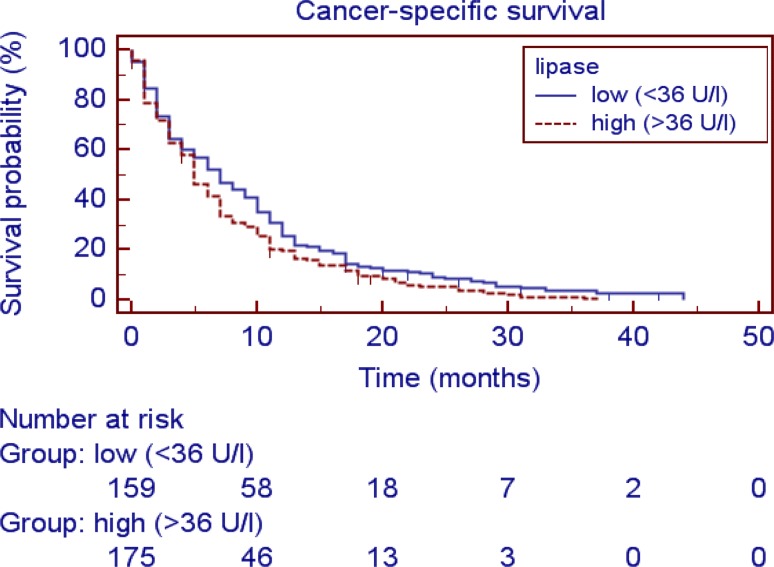
Kaplan Meier curve: Cancer specific survival: elevated Lipase vs. non elevated Lipase

In the study cohort a significant and strong correlation (Spearman correlation) between lipase and amylase levels (R = 0.821, *p* < 0.001) was found as well as between amylase and creatinine (R = 0.132, *p* = 0.015) but no significant correlation between amylase and CA19-9 (R = −0.04, *p* = 0.474) or age (R = −0.046, *p* = 0.398). Regarding the lipase levels, no correlation could be found between lipase and CA19-9 (R = −0.09 *p* = 0.108), age (R = −0.33, *p* = 0.543) or creatinine (R = 0.105, *p* = 0.050). There was also no correlation between amylase and the inflammatory marker Neutrophil-to-Lymphocyte-Ratio (NLR; R = 0.040, *p* = 0.587), lymphocyte count (R = 0.018, *p* = 0.768) or C‑reactive protein (R = 0.039, *p* = 0.536) and no correlation between lipase and the inflammatory marker NLR (R = 0.072, *p* = 0.324), lymphocyte count (R = 0.048, *p* = 0.438) or the C‑reactive protein (R = 0.039, *p* = 0.530). Furthermore, no association was found between pancreatic enzymes and gender, tumor grade, administration of gemcitabine-based chemotherapy or Karnofsky performance status (Tables 2 and 3). Applying receiver operating curve (ROC) analysis an optimal cut-off level was calculated for amylase at 20 U/l and for lipase at 36 U/l for further prognostic exploration. Fig. 1 shows the Kaplan-Meier curves for CSS and reveals that elevated levels of amylase are a consistent factor for poor prognosis in PC patients (*p* = 0.0288, log-rank test). Similarly, high levels of plasma lipase represent a poor prognostic factor (*p* = 0.0287, log-rank test, Fig. 2). To investigate whether the pancreatic enzymes and other clinicopathological factors are associated with the clinical outcome of PC patients, univariate and multivariate Cox proportional models for CSS were calculated. Univariate Cox proportional analysis further identified administration of chemotherapy (no treatment versus chemotherapy, *p* < 0.001), an elevated CA19-9 level (*p* = 0.001), amylase (>20 U/l versus ≤20 U/l, *p* = 0.047), lipase (>36 U/l versus ≤36 U/l, *p* = 0.039) and Karnofsky performance status (<80 versus 90–100, *p* = 0.001) as prognostic factors for poor CSS in this study cohort; however, age (>65 years versus ≤65 years, *p* = 0.412) was not significantly associated with clinical outcome (Table 4). To determine the independent prognostic value of amylase and lipase for CSS, a multivariate analysis using a Cox proportional hazard model was performed. In the multivariate analysis that included age, gender, the Karnofsky performance status and all factors significantly associated with survival in univariate analysis, high levels of CA19-9, administration of chemotherapy and high levels of amylase were identified as independent prognostic factors for CSS (HR = 1.373; 95% CI = 1.004–1.878; *p* = 0.047; Table 4).

**Table 2:** Relationship between clinicopathological parameters and the pretreatment plasma amylase of patients with stage IV pancreatic adenocarcinoma (*n* = 371)

Characteristics	Amylase <20 U/l	Amylase ≥20 U/l	*p*-value
*Age at diagnosis (years)*			
<65	64	76	0.435
≥65	101		
*Gender*			
Female	70	80	0.663
Male	95	97	
*Karnofsky index*			
<90	31	98	0.204
≥90	39	174	
*CA-19-9, U/ml*			
<Median	62	71	1.000
>Median	85	97	
*Chemotherapy*			
No	44	57	0.286
Yes	121	119

**Table 3:** Relationship between clinicopathological parameters and the pretreatment plasma lipase of patients with stage IV pancreatic adenocarcinoma (*n* = 371)

Characteristics	Lipase <36 U/l	Lipase ≥36 U/l	*p*-value
*Age at diagnosis (years)*			
<65	103	118	0.634
≥65	64	66	
*Gender*			
Female	77	75	0.33
Male	90	109	
*Karnofsky index*			
<90	64	66	0.634
≥90	103	118	
*CA-19-9, U/ml*			
<Median	62	73	0.734
>Median	90	96	
*Chemotherapy*			
No	46	58	0.414
Yes	121	125

**Table 4:** Univariate and multivariate Cox proportional analysis regarding cancer-specific survival

Parameter	Univariate analysis		Multivariate analysis	
	HR (95% CI)	*p*-value	HR (95% CI)	*p*-value
*Age at diagnosis(years)*				
Continuous rise per year	0.98 (0.99–1.02)	0.412	0.93 (0.73–1.19)	0.574
*Gender*				
Female	1 (reference)	0.488	1 (reference)	0.248
Male	1.07 (0.84–1.34)		1.15 (0.90–1.45)	
*Chemotherapy*				
No	1 (reference)	<0.001	1 (reference)	<0.001
Yes	0.32 (0.242–0.398)		0.32 (0.24–0.43)	
*CA19-9 elevated*				
No	1 (reference)	0.001	1 (reference)	0.035
Yes	1.46 (1.16–1.83)		1.29 (1.02–1.64)	
*Amylase*				
<20 U/l	1 (reference)	0.039	1 (reference)	0.034
≥20 U/l	1.29 (1.01–1.57)		1.40 (1.03–1.92)	
*Lipase*				
<36 U/l	1 (reference)	0.039	1 (reference)	0.689
≥36 U/l	1.38 (1.01–1.56)		0.94 (0.69–1.23)	
*Karnofsky index*				
<80	1 (reference)	<0.001	1 (reference)	0.314
90–100	0.67 (0.54–0.84)		0.88 (0.69–1.13)	
Creatinine (continuous)	1 (reference) 1.00 (0.99–1.02)	0.483	1 (reference) 1.01 (0.97–1.02)	0.176

*HR* hazard ratio, *CI* confidence interval

## Conclusion

Regarding the management of metastatic PC, there are only few measures with meaningful impact on the prediction of the survival time of patients. To a great extent an individual patient’s prognosis depends not only on the histology of the tumor but also on the extent of its spread (TNM stage). The ductal adenocarcinoma is the most common histological subtype and the prognosis is very poor. [15]. To keep invasive methods for determination of molecular and genetic prognostic factors to a minimum, circulating biomarkers may offer a promising area for further investigation [16, 17]. In the present study it could be verified that there is an association between elevated amylase at the time of diagnosis and poor clinical outcome for patients with metastatic PC.

Amylase has several isoforms, which can be secreted by the pancreas (P form) and the salivary glands (S form) but can also be found in smaller quantities in some other tissue types [18]. Amylase is a relatively small molecule (50,000 Daltons) with the main function of breaking down starch into smaller polysaccharides at the internal 1–4 alpha linkage during the digestive process [19]. The measurement of amylase in plasma is a commonly used routine test, obtained as a biomedical marker for acute pancreatitis [20]. There are several other reasons for elevated plasma amylase besides acute pancreatitis. For example, renal failure or more precisely impaired renal clearance may cause an increase of amylase in the bloodstream as soon as the creatinine clearance drops below 50 ml/min [21]. Therefore, a correlation analysis of amylase/lipase and markers of the systemic inflammatory response (CRP, NLR and lymphocyte count) was performed but no significant correlation between these markers could be found; however, a correlation was found between the creatinine level and the amylase level. It can be assumed that PC patients with high creatinine levels might not be the healthiest patients in general, which could also have an impact on the overall survival.

The most commonly used amylase assays cannot separate between pancreatic and salivary amylase. Alcoholics for instance may have increased amylase levels of salivary origin, which could create severe difficulties in interpretation when alcoholic abuse is not recorded in the patient’s anamnesis [22]. Plasma amylase may also be elevated when amylase is bound to immunoglobulin or polysaccharides forming the so-called macroamylase, which due to its larger size can no longer be filtered by the kidney anymore [23]. Under such circumstances patients typically present with a chronic elevation of amylase. To sum up, the predictive value of plasma amylase is crucially influenced by the clinical context; however, acute pancreatitis is not the only cause.

To return to amylase in association with cancer the question arises why patients with metastatic PC and elevated amylase levels have a poor prognosis. As PC, especially when located in the corpus or cauda of the pancreas, remains symptom-free for a considerable period of time, in most cases it has already formed distant metastases at time of diagnosis. It poses a big challenge to determine which of the affected organs is responsible for the elevation of amylase. Whether elevated levels of this biomarker are caused by liver failure due to massive liver metastases, by renal failure due to drugs or prior infections that damaged the kidneys or by the pancreas itself due to co-infection is unclear in the majority of cases. Therefore, one can only speculate about the pathophysiological background underlying these prognostic findings of amylase at the time of diagnosis, as data regarding any comorbidity that could have additionally affected the plasma amylase, were limited. Nonetheless, it could be demonstrated that an elevated amylase is associated with a significantly shorter cancer survival time in multivariate analysis.

Based on these findings, the non-invasive measurement of amylase levels at time of PC diagnosis seems to be a new marker for individual patient risk assessment and can be regarded as a promising independent prognostic parameter in PC patients.

The strength of this study is the large sample size and the long follow-up period; however, due to the retrospective design of the study a selection bias in the study cohort cannot be fully excluded. Even though the prognostic value of amylase determination in daily practice is perhaps limited as these significant results are only explored in advanced tumor stages and because the prognosis of the survival duration of PC patients is poor in general.

In conclusion, this study is the first to indicate that increased amylase levels are a negative prognostic marker in PC patients.

### Take home message

There are only few measures with meaningful impact on the prediction of the survival time of PC patients; however, circulating biomarkers tend to represent a promising area for investigation. In the present study it could be verified that there is an association between elevated amylase at the time of diagnosis and poor clinical outcome for patients with metastatic PC. The measurement of amylase in plasma is a commonly used routine test, which means it is a low cost, easy and non-invasive option to stratify patients into different risk groups in clinical trials and clinical decision-making.
